# Three-Dimensional Autofocusing Visual Feedback for Automated Rare Cells Sorting in Fluorescence Microscopy

**DOI:** 10.3390/mi10090567

**Published:** 2019-08-27

**Authors:** Huaping Wang, Kailun Bai, Juan Cui, Qing Shi, Tao Sun, Qiang Huang, Paolo Dario, Toshio Fukuda

**Affiliations:** 1 Beijing Advanced Innovation Center for Intelligent Robots and Systems, Beijing Institute of Technology, Beijing 100081, China; 2 Intelligent Robotics Institute, School of Mechatronical Engineering, Beijing Institute of Technology, Beijing 100081, China; 3 Key Laboratory of Biomimetic Robots and Systems (Beijing Institute of Technology), Ministry of Education, Beijing 100081, China; 4 Biorobotics Institute, Scuola Superiore Sant’Anna, Viale Rinaldo Piaggio 34, 56025 Pontedera, Pisa, Italy

**Keywords:** micromanipulation, visual feedback, autofocusing, rare cells sorting, fluorescence microscopy

## Abstract

Sorting rare cells from heterogeneous mixtures makes a significant contribution to biological research and medical treatment. However, the performances of traditional methods are limited due to the time-consuming preparation, poor purity, and recovery rate. In this paper, we proposed a cell screening method based on the automated microrobotic aspirate-and-place strategy under fluorescence microscopy. A fast autofocusing visual feedback (FAVF) method is introduced for precise and real-time three-dimensional (3D) location. In the context of this method, the scalable correlation coefficient (SCC) matching is presented for planar locating cells with regions of interest (ROI) created for autofocusing. When the overlap occurs, target cells are separated by a segmentation algorithm. To meet the shallow depth of field (DOF) limitation of the microscope, the improved multiple depth from defocus (MDFD) algorithm is used for depth detection, taking 850 ms a time with an accuracy rate of 96.79%. The neighborhood search based algorithm is applied for the tracking of the micropipette. Finally, experiments of screening NIH/3T3 (mouse embryonic fibroblast) cells verifies the feasibility and validity of this method with an average speed of 5 cells/min, 95% purity, and 80% recovery rate. Moreover, such versatile functions as cell counting and injection, for example, could be achieved by this expandable system.

## 1. Introduction

Low-abundance cells with samples containing less than 1000 target cells/ml are considered as rare cells [1]. Research on sorting and isolating a specified number of rare cells, such as circulating tumor cells (CTCs), circulating fetal cells, and stem cells from complex and heterogeneous mixtures, are vitally important in biology and medicine [2]. This practice often serves as the preparatory work in many diagnostic and therapeutic practices to enhance the research efficiency [3]. CTCs, for example, are recognized as the biomarker for various cancers, such as breast, prostate, ovarian, colon, etc. [4,5]. Sorting and detecting CTCs from cancer patients is viable for cancer prognosis and treatment. Meanwhile, the rapid and accurate sorting of rare cells is highly demanded in personalized medicine. The tailored medicine improves the precision and effectiveness of therapy [6].

Various methods have been proposed for cell sorting. Density-gradient centrifugation (DGC) separates targets by the density difference between cell types [7]. However, the recovery rate of the desired cell is largely reduced, and undesired cell types are introduced as heterogeneous cells [8]. Fluorescence-activated cell sorting (FACS), as the first commercial cell sorter, was invented in 1969 [9]. As a high-throughput system, FACS detects and analyzes multiple cell types with sorting speed up to 50,000 cells per second [10]. However, FACS is limited for clinical application and commercialization due to the high initial costs of system, the risk of sample contamination, and the necessity of technical expertise for operating complex machinery [10]. Another commercially available sorter, the immunomagnetic-assisted cell sorting (IMACS) system, was invented later. It is capable of selecting circulating tumor cells, fetal cells, and stem cells at a high recovery rate of about 80% [11]. However, the magnetic field used in IMACS may produce perturbations on cell differentiation, which the viability of cells is affected by to some extent [1]. In recent years, microfluidic cell sorting was presented as a new approach [3]. Through combining with FAC, IMACS and wireless driven forces derived from magnetic, electric, and optical fields, it provides numerous advantages over conventional methods such as small equipment size and low reagent consumption [12,13,14,15,16,17,18]. However, the limited lifespan of microfluidic chips and complex sample preparations are barriers for clinical use. Unfortunately, most of the existing rare cells sorting methods are non-automated or semiautomated, where operators are required. The misoperation and fatigue of operators can lead to the unreliable and unrepeatable experimental data.

Bio-micromanipulation, driven by micro-nano technology and biomedical engineering, provides a new approach to rare cells sorting. As a straightforward method, bio-micromanipulation is capable of performing a series of operations such as injection, aspiration, and gripping [19,20,21]. However, it is generally performed manually or is semiautomated with poor repeatability and success rate. The combination of bio-micromanipulation and robots has generated considerable research interest recently [22,23,24,25,26]. To increase the accuracy and realize automated bio-micromanipulation, visual-servo control is necessary for microrobotic manipulation [27]. A semi-automated robot was reported for automated single cell isolation from suspension with visual-servo control [28]. Whereas only cells’ planar locations are provided as the lack of depth limits the application of robots in thin-layer suspensions. Cell depth is the basis for the three-dimensional (3D) location of cells, and subsequently automated sorting. To obtain the depth of targets, Mulligan reported a fast calibrated stereo vision for micromanipulation [29]. Li et al. described a dual-camera system which is mounted on a stereo light microscope to achieve 3D displacement measurement at microscale, where enough depth of filed (DOF) is essential [30]. However, most microscopes are featured with shallow DOF, which makes an efficient visual-processing-based method for the 3D detection of objects a great necessity. In addition, the clearness of targets, especially for rare cell sorting, is critical to accurate 3D information capture. Most of the micromanipulation systems work under a bright-field illumination microscope. The low contrast between cells and background increases the difficulty of detection, and the overlap of multiple cells reduces the precision of the location. Compared with its traditional counterpart, the fluorescence microscope has a better performance of clearness superiority over bright field [31]. Fluorescence, as a non-invasive label, maintains cell viability and makes visual processing easy [32]. Nevertheless, the time of fluorescence observation is limited due to the fluorescence quenching [33]. The fast visual processing algorithm for rare cell fluorescence observation is lacking.

**Figure 1:**
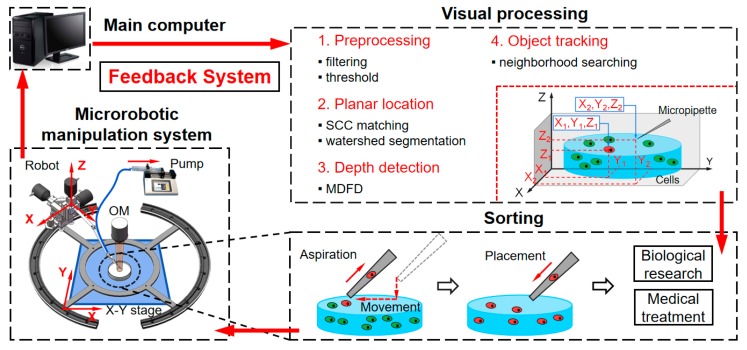
Schematic of automated sorting process.

In this paper, we propose a fast autofocusing visual feedback (FAVF) method for automated sorting of rare cells under fluorescence microscope. The whole sorting process is shown in Figure 1. The microrobotic manipulation system is mounted in the inverted fluorescence microscope. The fluorochrome enhances the clarity and contrast of cells and micropipette clear. The visual information is obtained by a camera and transmitted to the main computer simultaneously. In visual processing, the FAVF algorithm mainly consists of four parts: preprocessing, planar locating, depth detection, and object tracking. Planar locating relies on the scalable correlation coefficient (SCC) matching and watershed segmentation algorithm. Depth detection is achieved by the improved multiple depth from defocus (MDFD) algorithm, which derives from traditional depth from defocus (DFD). The neighborhood search based algorithm is adopted for object tracking. Assisted by FAVF, real-time 3D locating of micropipette and cells are achieved. With the visual feedback, automated rare cells sorting is carried out by microrobotic system with high positioning accuracy and high operating efficiency. The system is evaluated by sorting DiI (cell membrane red fluorescent probe) stained NIH/3T3 cells with red fluorescence from DiO (cell membrane green fluorescent probe) stained NIH/3T3 cells with green fluorescence. The experiment demonstrates the system has considerable sorting speed, purity and recovery rate. Due to its simplicity, durability, cost-effectiveness and expandability, the system is viable for the isolation of most rare cell types and promising for biological and medical research.

## 2. Materials and Methods

### 2.1. Planar Locating

#### 2.1.1. Scalable Correlation Coefficient Matching

Template matching algorithm has been widely used in micromanipulation for its accurate and fast performance. For cell sorting, target cells are scattered in suspension with different spatial distributions. Due to the shallow DOF of microscope, cells have various sizes and the degrees of blur. In traditional template matching method, template is preselected with fixed size, which is inapplicable in this case. The scalable correlation coefficient (SCC) matching algorithm for planar locating is proposed. According to the correlation coefficient equation:

(1) $$R(x,y)=\sum_{x^{\prime },y^{\prime }}\left(T^{\prime }(x^{\prime },y^{\prime })\cdot I^{\prime }(x+x^{\prime },y+y^{\prime })\right)$$

and

(2) $$T^{\prime }(x^{\prime },y^{\prime })=T(x^{\prime },y^{\prime })-1/(w\cdot h)\cdot \sum_{x^{\prime \prime },y^{\prime \prime }}T(x^{\prime \prime },y^{\prime \prime })$$

(3) $$I^{\prime }(x+x^{\prime },y+y^{\prime }))=I(x+x^{\prime },y+y^{\prime })-1/(w\cdot h)\cdot \sum_{x^{\prime \prime },y^{\prime \prime }}I(x+x^{\prime \prime },y+y^{\prime \prime })$$

where *x* and *y* are coordinate value of pixels, *T* is grayscale value of template, *I* is grayscale value of source image, *w* and *h* are the width and height of template, *R* is correlation coefficient. The correlation coefficient value and matching degree are positively correlated.

**Figure 2:**
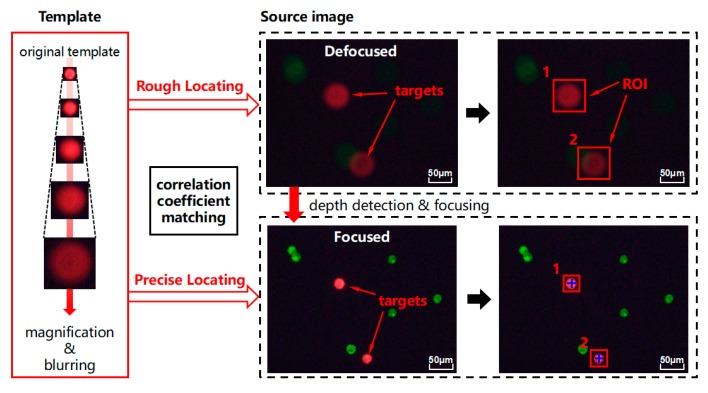
Schematic of scalable correlation coefficient matching.

As shown in Figure 2, focused cell is preselected as the original template. It is enlarged to a series of templates with Gaussian blur. In rough locating, two blurred target cells are detected by scalable templates with optimal sizes and are marked by a bounding box. The result of rough locating generates the region of interest (ROI) for subsequent depth detection. The focused target cells are matched by optimal template and their precise locations are marked with tow cross lines.

#### 2.1.2. Watershed Algorithm Based Instance Segmentation

**Figure 3:**
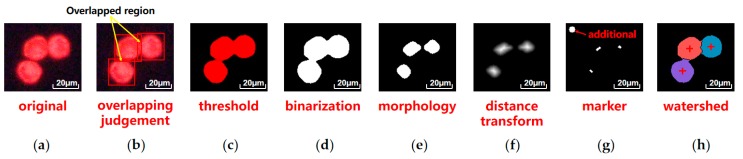
Process of watershed algorithm based instance segmentation: (**a**) Original cell image; (**b**) Overlapping judgement by detecting the overlapped region of the bounding boxes; (**c**) Red color channel is remained by setting threshold; (**d**) Binarization with the Otsu adaptive threshold method; (**e**) Morphology erosion and dilation operation to separate cells; (**f**) Distance transform to refine regions; (**g**) Markers generation by threshold and find contours; (**h**) Targets separation by watershed algorithm.

In the microscopic scene, cells are easily overlapped, due to the electrostatic force and adhesive force. Instance segmentation of target cells is essential for precise locating. The newly developed deep learning algorithm provide satisfactory results. However, data collecting and training is time consuming. Here, we present a watershed algorithm-based instance segmentation method. The process of this method is shown in Figure 3. Three target cells are stained red and stuck together (Figure 3a). The overlapped cells are sketchily marked with bounding boxes by SCC matching. In overlapping process, the presence of overlapping regions can trigger the subsequent segmentation (Figure 3b). Red color channel is remained by setting threshold (Figure 3c). Markers are created as water holes through a series of visual transforms (Figure 3g). An additional marker is set in the top left as background water hole. Markers are applied to Figure 3b through watershed algorithm, and boundary lines formed between cells and background. Target cells are segmented and their locations are marked by the center of the respective area (Figure 3h).

#### 2.1.3. Neighborhood Searching Based Micropipette Tracking

**Figure 4:**
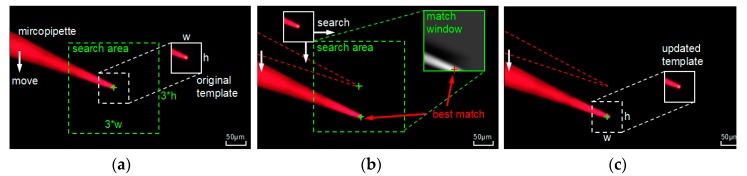
Process of neighborhood search based micropipette tracking: (**a**) Locating of micropipette tip and generating template; (**b**) Searching in the neighborhood and locating the new position of micropipette tip; (**c**) Updating match template for subsequent tracking.

To feed back the location of the micropipette tip in real-time, tracking algorithm is crucial. As shown in Figure 4a, when the micropipette gets focused, its tip is precisely located. Centering on the micropipette tip, the original tracking template and neighborhood searching area are created. To decrease the running time of program, the size of neighborhood searching area is limited to three times the size of the template. When the micropipette moves, as shown in Figure 4b, the original template searches in the neighborhood search area, and the match window is obtained by the correlation coefficient matching algorithm. The best match is indicated in the match window, and the location of micropipette tip is updated. Due to the fluorescence quenching and impurity adsorption, the color and shape of the micropipette can change slightly from the initial appearance. As shown in Figure 4c, the updated template has better performance to adapt subsequent tracking.

### 2.2. Depth Detection

Autofocus technology is one of the key technologies in computer vision and various imaging systems. It has a wide range of applications such as cameras, endoscopes and microscopes. In microscope imaging, depth from focus (DFF) and depth from defocus (DFD) are two widely used methods, which are essential for the acquisition of depth value and clear observation [21,34]. The DFD method has a great detection efficiency by comparing two defocused images. In this paper, to solve the symmetrical problem in traditional DFD, the multiple depth from defocus (MDFD) method is presented.

#### 2.2.1. Imaging Model of Multiple Depth from Defocus (MDFD)

**Figure 5:**
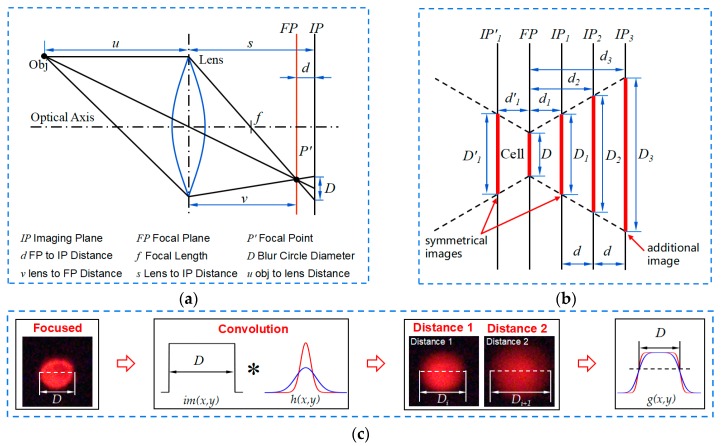
Theory of multiple depth from defocus (MDFD): (**a**) convex lens imaging model; (**b**) imaging model for cell; (**c**) theory of point spread function.

The convex lens imaging model of MDFD shown in Figure 5a. Through the lens, the object is focused as a focal point (*P’*) on the focal plane (*FP*). Adjusting the imaging plane (*IP*) away from *FP*, a defocused blur circle formed, where *d_n_* is the object depth value. As shown in Figure 5b, in the imaging model, a cell is focused on the focal plane with focused diameter *D*. Changing *IP* successively with a fixed step interval *d*, and the blur diameter *D_n_* acts like a linear shift. According to geometry:

(4) $$d_{n}=\left(\frac{D_{n}-D}{D_{n+1}-D_{n}}\right)d$$

where object depth value *d_n_* is obtained when *D* and *D_n_* are known. The point spread function (PSF, *h(x,y)*) is introduced to explain this phenomenon—it is modeled as two dimensional Gaussian:

(5) $$h(x,y)=\frac{1}{2\pi \sigma^{2}}\exp\left(-\frac{x^{2}+y^{2}}{2\sigma^{2}}\right)$$

where spread parameter σ is positive correlated to *D_n_*. As shown in Figure 5c, the observed image g(x,y) is the result of the convolution between the focused image im(x,y) and PSF:

(6) g(x,y)=h(x,y)∗im(x,y)

When *IP* moves away from *FP*, brightness energy of the cell is dispersed and the observed *D_n_* is getting larger. For a certain cell, the energy distribution curves on different *IP* have the same two intersections. The width of two intersections is the focused diameter *D* of the cell.

However, the symmetrical problem exists. As shown in Figure 5b, in symmetrical position (*IP_1_* and *IP’_1_*) on either side of the plane, objects have the same blur diameter (*D’_1_* and *D_1_*). They cannot be distinguished by the traditional two-image DFD method. 

#### 2.2.2. Algorithm Strategy of Multiple Depth from Defocus (MDFD)

**Figure 6:**
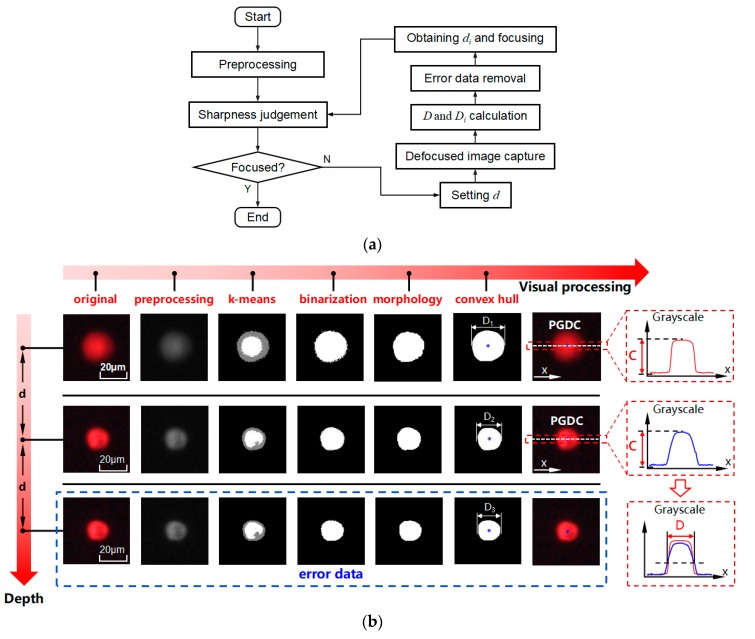
The theory of multiple depth from defocus (MDFD): (**a**) Flow chart of MDFD; (**b**) Visual processing of MDFD.

To solve the symmetrical problem, MDFD captures additional images and possesses optimized performance. The flow chart of MDFD is shown in Figure 6a, preprocessing and sharpness judgement of target cell are made in the region of interest (ROI). If the cell is defocused, setting a step interval *d* to capture a series of images along the z axis. *D* and *D_n_* are calculated by visual algorithm, and error data can be screened out by analysis the change of *D_n_*. The cell depth value *d_n_* is calculated by equation (4). The above process may be performed multiple times, until a target gets focused.

The visual processing of MDFD is shown in Figure 6b. Preprocessing consists of grayscale transformation and Gaussian filtering. The k-means clustering algorithm is applied to precisely separate the cell from background. Cluster number k is set to 3 to represent background, cell blur edge, and body respectively. Depending on the grayscale value, the pixels will be tagged with three different labels. According to labels, the regions of cell blur edge and body are combined to represent entire cell by image binarization. Morphological operation is applied to remove smaller areas smooth the cell contour. Cell contour is optimized by convex hull algorithm to search minimum area encirclement lines. Then cell center is obtained by calculation of image moments and blur diameters of cells *D_n_* are obtained. Extracting pixels across the horizontal line of the cell center (PAHLCC) from convex hull image. For grayscale images, the horizontal lines through the center of the objects are extracted and pixel grayscale distribution curves (PGDC) are obtained. Based on the PGDC, a sharpness judgement algorithm is proposed. For each PGDC, the sharpness of an image is evaluated by the difference between the maximum and minimum, which is considered as image contrast *c*. The larger the value of *c*, the clearer the image. According to the law of *c* change, error data (symmetrical case) is screened out. Besides, for different PGDC, the length of two intersections is focused diameter *D* of cell. Finally, *d* is calculated through Equation (4).

## 3. Results and Discussion

### 3.1. Experimental Setup

#### 3.1.1. System Framework

**Figure 7:**
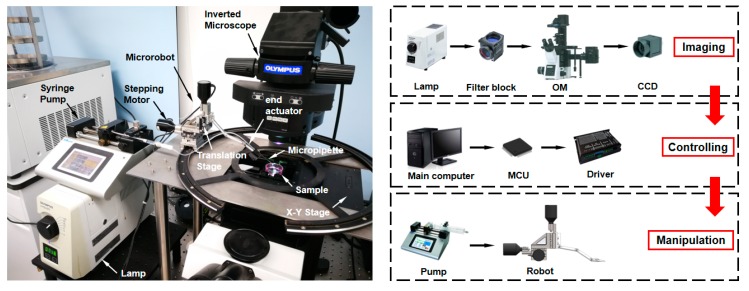
Automated microrobotic manipulation system.

The microrobotic manipulation system is shown in Figure 7. The robot consists of a linear translation stage (M-461-XYZ, Newport, Irvine, CA, USA) and three micro-stepping motors (NSA 12, Newport, Irvine, CA, USA) with a resolution of 0.2 µm and a motion range of 11 mm for each axis. A glass micropipette (B100-50-10, Sutter Instrument, Novato, CA, USA) is heat pulled by a micropipette puller (PC-10, Narishige, Tokyo, Japan) with an inner diameter of 19 μm. It is fixed on an end actuator and connected to the syringe pump (Legato 111, KD Scientific, Ringoes, NJ, USA) via a soft rubber tube. An inverted optical microscope (OM) (IX83, Olympus, Tokyo, Japan) is connected to a CCD camera (DP22, Olympus, Tokyo, Japan) with maximum 2.76 megapixels. The lamp (U-HGLGPS, Olympus, Tokyo, Japan) and filter block (U-FNU/FNB/FNG, Olympus, Tokyo, Japan) ensure multi-colored fluorescence observation. A motorized X-Y translational stage (ProScan, Prior Scientific, Cambridge, UK) mounted on the OM can transform field of view (FOV) rapidly. A computer configured with CPU (Core i7, Intel, Santa Clara, CA, USA) and GPU (TITAN X, NVIDIA, Santa Clara, CA, USA) is utilized for visual processing and automated control.

#### 3.1.2. Experiment Preparation

**Figure 8:**
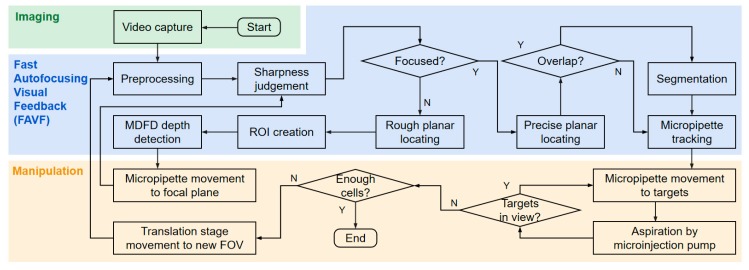
Overall process of automated cell sorting.

NIH/3T3 (ATCC, Manassas, VA, USA) cells are chosen as sample due to their stable activity and morphology in vitro culture. Cells stained with DiI (red)(beyotime, shanghai, china) and DiO (green)(beyotime, shanghai, china) are mixed in a proportion of 1:100. The heterogeneous mixture is composed of approximate 100 mL^−1^ red target cells and 10,000 mL^−1^ green cells. Cells were suspended in culture medium consists of Dulbecco’s Modified Eagle’s Medium (DMEM, Hyclone, Logan, UT, USA) supplemented with 10% (v/v) fetal bovine serum (FBS, Gibco, Gaithersburg, MD, USA) and 1% (v/v) penicillin-streptomycin solution (Solarbio, Beijing, China). Furthermore, the micropipette tip is pre-dyed with DiI. Under the fluorescence microscope, the red and green lights are excited. The overall process of automatic microrobotic manipulation strategy is shown in Figure 8. The camera transmits microscopic images to the main computer for visual processing. For FAVF, impurities filtering and images smoothing are carried out in preprocessing. Sharpness judgement distinguishes blurred and focused targets. The depth value of the micropipette tip and target cells are obtained through the MDFD method. According to the depth value, the micropipette moves to the same focal plane as the target cells. Then, the micropipette and target cells are located by precise planar locating algorithm and the overlapped targets are separated by segmentation algorithm. During the manipulation, the position of micropipette tip is tracked simultaneously. With the visual feedback, robots are controlled to move and aspirate target cells until all are screened in the FOV. The new group of cells are brought into the FOV by the translation stage. Sorting will continue until the number of cells collected is sufficient.

### 3.2. Planar Locating and Tracking

#### 3.2.1. Planar Locating

**Figure 9:**
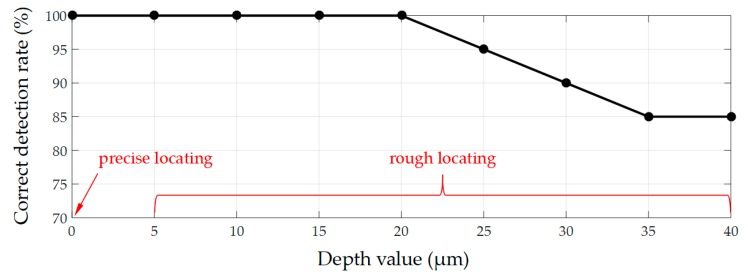
Correct detection rate of planar locating.

The scalable correlation coefficient (SCC) matching algorithm were adopted for both rough locating and precise locating. The algorithm was evaluated at 9 groups of depth value. For each group, 20 targets were tested. A target should be detected with the correct number and location, otherwise it will be considered as an error detection. As shown in Figure 9, the correct detection rate is 100% in the precise locating section. In the rough locating section, the correct detection rate slightly declines when the depth value exceeded 20 µm. The average correct detection rate is 95% and the average processing time is 0.8 s, which guarantees real-time feedback.

#### 3.2.2. Micropipette Tip Tracking

**Figure 10:**
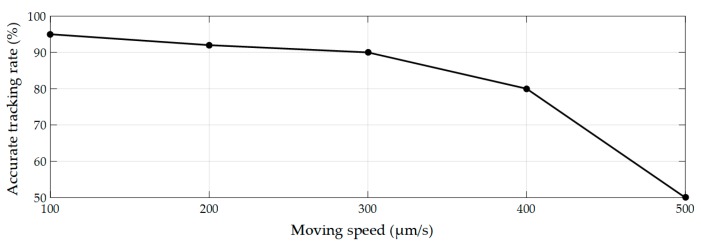
Accurate tracking rate for micropipette tracking.

The neighborhood search based tracking algorithm was used for micropipette tip tracking. The algorithm was evaluated by the movement of the micropipette at five groups of speed. The CCD camera has a frame rate of 60 fps and 1500 frames were tested at each group. The location of micropipette tip was marked by bounding box in each frame of image. Between consecutive frames, the ratio of overlapped region of the bounding boxes is defined as overlapping rate. When overlapping rate is greater than 70%, it is considered as accurate tracking. As shown in Figure 10, the accurate tracking rate drops significantly when speed is over 300 µm/s. To ensure the tracking accuracy, the moving speed of micropipette was set to 300 µm/s, which allowed a high accuracy (90%) and a sorting speed of 2 s/cell.

### 3.3. Depth Detection with Multiple Depth from Defocus (MDFD)

**Figure 11:**
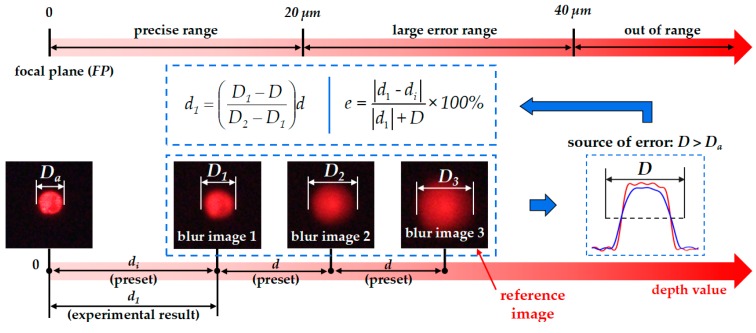
Schematic of multiple depth from defocus (MDFD) experiment.

**Figure 12:**
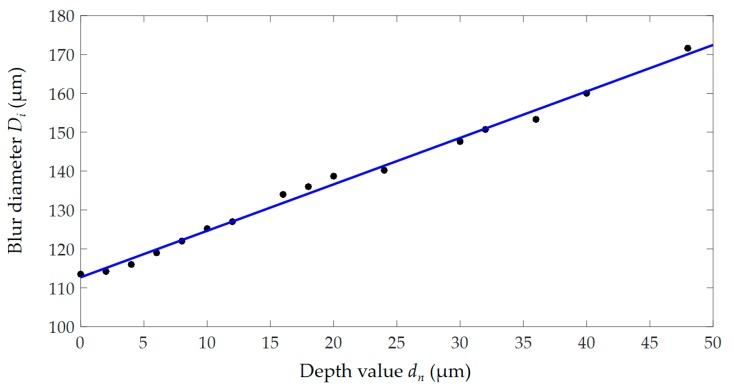
Linear distribution of *d_n_* and *D_n_* for 1 out of 10 samples.

For the sample cell, the blur diameters (*D_n_*) on different image planes (*IP*) were calculated by previously mentioned algorithm (Figure 6b). To verify the reliability of *D_n_*, the depth value of focal planes (*FP*) was set to 0, which was determined by sharpness judgement algorithm. The diameters of cells on the *FP* was considered as the actual focused diameter (*D_a_*), as shown in Figure 11. The *IP* was adjusted at unequal step intervals (*d*) and *D_n_* were obtained. As shown in Figure 12, scatter plot for depth values and *D_n_* was drawn and one-time polynomial was applied for data fitting. A total of 10 sample cells were tested in the experiment and the result showed that the average coefficient of determination (R-square) is 0.9953. It indicated that scatter points are generally linear and the algorithm for *D_n_* detection was almost feasible.

Then, to evaluate the accuracy of depth detection of MDFD for the sample cell, the *IP* was set to a known initial depth (*d_i_*), as shown in Figure 11. Moreover, each *IP* was adjusted at a certain *d* with the image recorded. The experimental depth value (*d_n_*) was calculated by Equation (4). To evaluate the deviation of this method, the relative error formula is presented:

(7) $$e=\frac{\left|d_{n}-d_{i}\right|}{\left|d_{n}\right|+D}\times 100\%$$

where *e* is the experimental error. The experimental focused diameter (*D*) is introduced as correction factor. 

**Figure 13:**
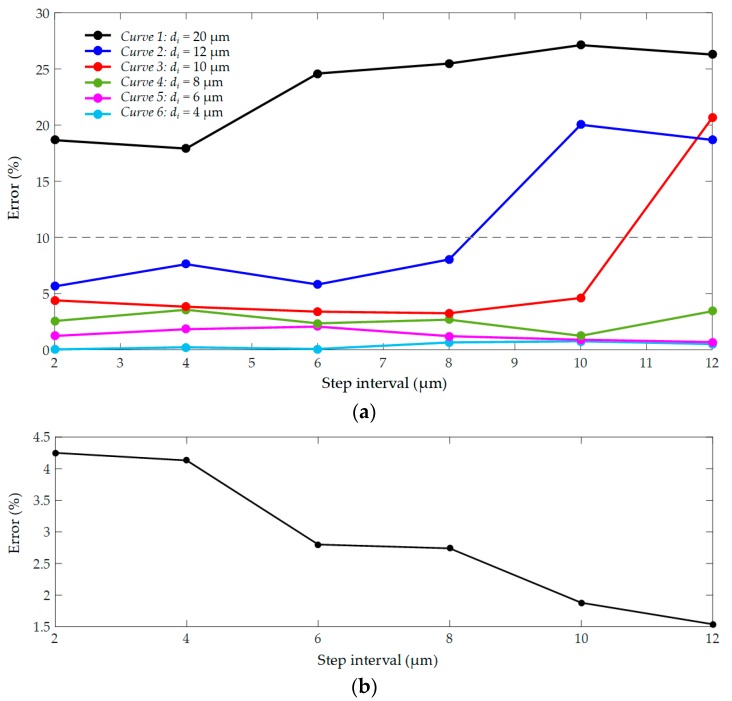
Error of multiple depth from defocus (MDFD): (**a**) Error at different initial depths; (**b**) Error at different step intervals.

Set *d_i_* from 4 mm to 36 mm and *d* from 2 mm to 12 mm, respectively. When the depth value is more than 40 µm, the fluorescence is very weak which cannot be recognized. Therefore, the valid test data *d_n_* are limited within the range of 40 µm, otherwise it is considered as out of range (OOR). A total of 10 sample cells were tested in the experiment, and the average *e* are shown in Table 1. According to the table data, error curve for different *d_i_* were drawn, as shown in Figure 13a. In curve 4, 5 and 6, *e* is all lower than 5%. However, in curve 2 and 3, *e* increases sharply and exceeds 10% at *d* of 10 µm and 12 µm, respectively. In curve 1, the average *e* is greater than 20%. The data showed that when *d_n_* are within 20 µm the results are highly precise. However, when *d_n_* exceeds 20 µm, the result is unreliable. Such a large deviation was derived from the calculation of *D*. Due to the imaging noise, *D* was always larger than *D_a_* which is inevitable. Then, multiple calculations were applied, as shown in Figure 11, and the depth values can finally converge to be accurate. 

In a precise range, *e* can decrease significantly when *d* is greater than 4 µm, as shown in Figure 13b. However, too large *d* can make *d_n_* into the large error range and it was set to 6 µm. To solve the symmetry problem, the algorithm worked based on the three blur images, and one of them was used as a reference. The results showed that the depth values were obtained after approximately two or three calculations with an almost small error of 3.21%. The entire focusing process took a short 850 ms averagely and the real-time feedback is guaranteed.

### 3.4. Automated Fast Autofocusing Visual Feedback (FAVF) Cell Sorting

**Figure 14:**
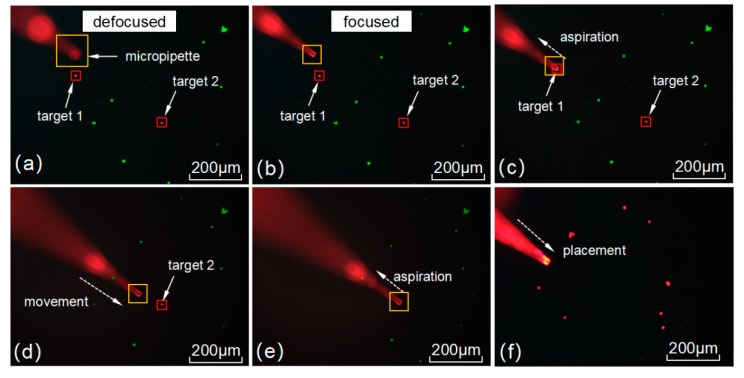
Automated fast autofocusing visual feedback (FAVF) cell sorting.

**Figure 15:**
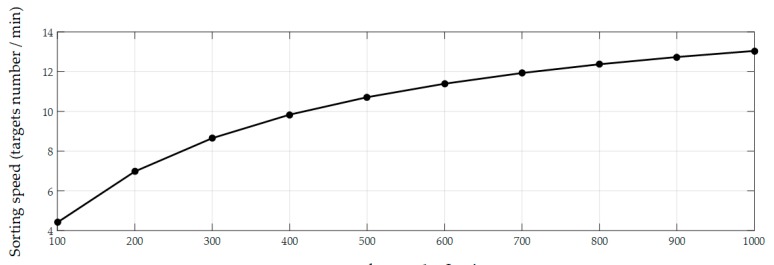
Plot of sorting speed versus different target number.

The performance of the FAVF cell sorting method was evaluated using heterogeneous mixtures under the fluorescence microscope. The volume of the mixture was 1 mL and 100 target NIH/3T3 cells were contained. The rough planar locations of micropipette and target cells were detected by SCC matching algorithm (Figure 14a). FOV was focused after acquiring depth by MDFD and precise planar locations were obtained (Figure 14b). The micropipette was controlled to move to target cells with tip was tracked. When all targets were sorted, the FOV was transformed by regular scanning on the surface of a Petri dish with a diameter of 35 mm. Scanning stopped until the sufficient number of cells was collected (see Supplementary Video S1). The result of 10 experiments showed that the proposed method achieved the average speed of 5 cells/min, a purity of target cells of 95%, and recovery rate of 80%. The introduction of a small number of heterogeneous cells is mainly due to the deformation of the soft rubber tube—which is why a hard rubber tube will be utilized in the future research. In the entire process, FOV switching takes up the bulk of time and limits the sorting speed greatly. When additional target cells were added in the mixture, the speed got obviously increased, as shown in Figure 15. When the concentration was raised to 1000 target cells mL^−1^ and the sorting speed increased obviously. However, with an increase of target number, an upward trend gradually decreases in intensity. The optimal concentration was about 600 target cells/mL, with an average speed of 11 cells/min, where the sorting efficiency was mainly limited by the increment of computational complexity for 3D locating, segmentation and tracking. Meanwhile, FOV switching took approximately 10 min, scanning the entire surface of the Petri dish with a diameter of 35 mm. Therefore, the optimized the switch strategy and algorithm will be carried out in the future work to improve system performance. Cell viability before and after sorting was calculated based on statistic data by using calcein AM fluorescent stains (Molecular Probes, Eugene, OR, USA). Three independent experiments were carried out and the average cell viability were 98% (before sorting) and 90% (after sorting), which demonstrated the non-invasiveness of our method.

Compared with the DGC method, our system allows the markedly improved purity and recovery rate. Besides, this approach also shows some advantages over the FACS and IMACS methods such as a short time sample preparation, the requirement of convenient equipment, and non-invasive features. In addition, compared with microfluidic cell sorting, the robotic system is durable and reusable. Moreover, in comparison to traditional bio-manipulation methods, it realizes automated 3D locating with optimized depth detection algorithm without extra devices introduced. 

The proposed method is likely universalizable for the isolation of most rare cell types, such as CTCs, stem cells, and circulating fetal cells, which could make further contributions to medical diagnosis and tailored medicine. Moreover, the open-ended system means the addition of multiple visual algorithms and end actuators. It may achieve more functions like cell counting and injection for versatile applications across biology, biotechnology, and medicine.

## 4. Conclusions

In this study, a novel FAVF algorithm for the automated sorting of rare cells under the fluorescence microscope was proposed. The algorithm is performed on the microrobotic manipulation system with visual feedback control. In FAVF, the accurate 3D locations of objects are obtained in real time. SCC matching is presented for both rough and precise planar locating. The watershed segmentation algorithm is introduced to separate the overlapped cells. The improved MDFD algorithm is used in depth detection. The neighborhood search based algorithm is applied for tracking the movement of the micropipette. With visual feedback, robots are precisely controlled to move and aspirate target cells automatically. The experiment of screening NIH/3T3 cells demonstrates the good performance of the proposed system. Compared with other existing methods, this system has many advantages, such as relatively high purity and recovery rate, universalizability, simplicity, durability, and cost-effectiveness.

## Figures and Tables

**Table 1:** Error of multiple depth from defocus (MDFD) at different initial depth and step interval.

Initial Depth *d_i_*	*e* at *d* of 2 µm	*e* at *d* of 4 µm	*e* at *d* of 6 µm	*e* at *d* of 8 µm	*e* at *d* of 10 µm	*e* at *d* of 12 µm
4 µm	0.03%	0.22%	0.07%	0.65%	0.76%	0.51%
6 µm	1.23%	1.83%	2.06%	1.21%	0.89%	0.68%
8 µm	2.56%	3.56%	2.68%	2.35%	1.24%	3.43%
10 µm	4.40%	3.83%	3.39%	3.25%	4.61%	20.65%
12 µm	5.64%	7.62%	5.81%	6.23%	20.03%	18.68%
16 µm	7.26%	7.74%	21.56%	23.42%	20.58%	23.44%
18 µm	8.65%	23.64%	23.78%	21.56%	23.68%	23.81%
20 µm	18.66%	17.91%	24.59%	25.48%	27.12%	26.28%
24 µm	17.59%	16.23%	15.52%	11.07%	18.65%	18.39%
30 µm	27.58%	27.54%	27.17%	27.26%	28.96%	OOR
32 µm	20.54%	23.44%	18.65%	16.63%	OOR	OOR
36 µm	23.33%	22.84%	OOR	OOR	OOR	OOR

## Supplementary Materials

The following are available online at https://www.mdpi.com/2072-666X/10/9/567/s1, Video S1: Three-Dimensional Autofocusing Visual Feedback for Automated Rare Cells Sorting in Fluorescence Microscopy.
